# Popularity of internet physician rating sites and their apparent influence on patients’ choices of physicians

**DOI:** 10.1186/s12913-015-1099-2

**Published:** 2015-09-26

**Authors:** Christopher M. Burkle, Mark T. Keegan

**Affiliations:** Department of Anesthesiology, Mayo Clinic, Rochester, MN USA

## Abstract

**Background:**

There has been a substantial increase in the number of on-line health care grading sites that offer patient feedback on physicians, staff and hospitals. Despite a growing interest among some consumers of medical services, most studies of Internet physician rating sites (IPRS) have restricted their analysis to sampling data from individual sites alone. Our objective was to explore the frequency with which patients visit and leave comments on IPRS, evaluate the nature of comments written and quantify the influence that positive comments, negative comments and physician medical malpractice history might have on patients’ decisions to seek care from a particular physician.

**Methods:**

One-thousand consecutive patients visiting the Pre-Operative Evaluation (POE) Clinic at Mayo Clinic in Rochester Minnesota between June 2013 and October 2013 were surveyed using a written questionnaire.

**Results:**

A total of 854 respondents completed the survey to some degree. A large majority (84 %) stated that they had not previously visited an IPRS. Of those writing comments on an IPRS in the past, just over a third (36 %) provided either unfavorable (9 %) or a combination of favorable and unfavorable (27 %) reviews of physician interactions. Among all respondents, 28.1 % strongly agreed that a positive physician review alone on an IPRS would cause them to seek care from that practitioner. Similarly, 27 % indicated that a negative IPRS review would cause them to choose against seeking care from that physician. Fewer than a third indicated that knowledge of a malpractice suit alone would negatively impact their decision to seek care from a physician. Whether a respondent had visited an IPRS in the past had no impact on the answers provided.

**Conclusions:**

Few patients had visited IPRS, with a limited number reporting that information provided on these sites would play a significant role in their decision to seek care from a particular physician.

## Background

Although much research on patient satisfaction with their overall health care experience has been published recently, little information is available concerning patient satisfaction and experiences with individual physicians [1, 2]. Over the last decade, there has been a substantial increase in the number of on-line health care grading sites that offer patient feedback on physicians, staff and hospitals. These Internet-based health care rating sites are viewed as part of a larger enterprise focusing on consumer interests such as restaurants, hotels and plumbers, to name just a few. In one recent poll, almost a quarter of Internet users reported reading online reviews prior to purchasing goods or services offline, while fewer (14 %) reviewed medical service sites [3, 4]. However, among those using rating sites for medical services, over three quarters reported that the information gleaned had a significant influence on their purchase [3, 4]. Users in general voiced that reviews generated by fellow consumers had a greater influence than those generated by professionals [4].

Included among this growing industry of online health care service reviews are Internet physician rating sites (IPRS). In 2010, it was reported that 16 % of just over 3000 Internet users reported accessing online physician ranking or review sites in the past [5]. In 2010, it was estimated that one in six physicians practicing in the United States (U.S) has been reviewed online [6]. Despite a growing interest among some consumers of medical services, most studies of IPRS have restricted their analysis to sampling data from individual sites [2, 3, 6–8]. As a result, they are often limited to gathering information on the percentage of physicians rated and assessment of whether the ratings were positive or negative [3, 6–8]. In one recent study however, the influence these sites had on U.S. consumers’ choices when selecting a primary care physician was explored [9]. The study did not evaluate for differences between those who had, and had not, visited such sites. In addition, the study was conducted using an Internet-based survey instrument, potentially selecting a study population that was more “Internet savvy” and younger than consumers of healthcare in general.

In the present study we aimed to establish the frequency with which patients frequenting our institution visit and leave comments on IPRS, identify those sites most commonly visited, and evaluate the nature of comments written. In addition, we sought to quantify the influence that positive and negative comments along with information on a physician’s medical malpractice history might have on patients’ decisions to seek care from a particular physician. We stratified this feedback according to previous use of an IPRS.

## Methods

**Fig. 1:**
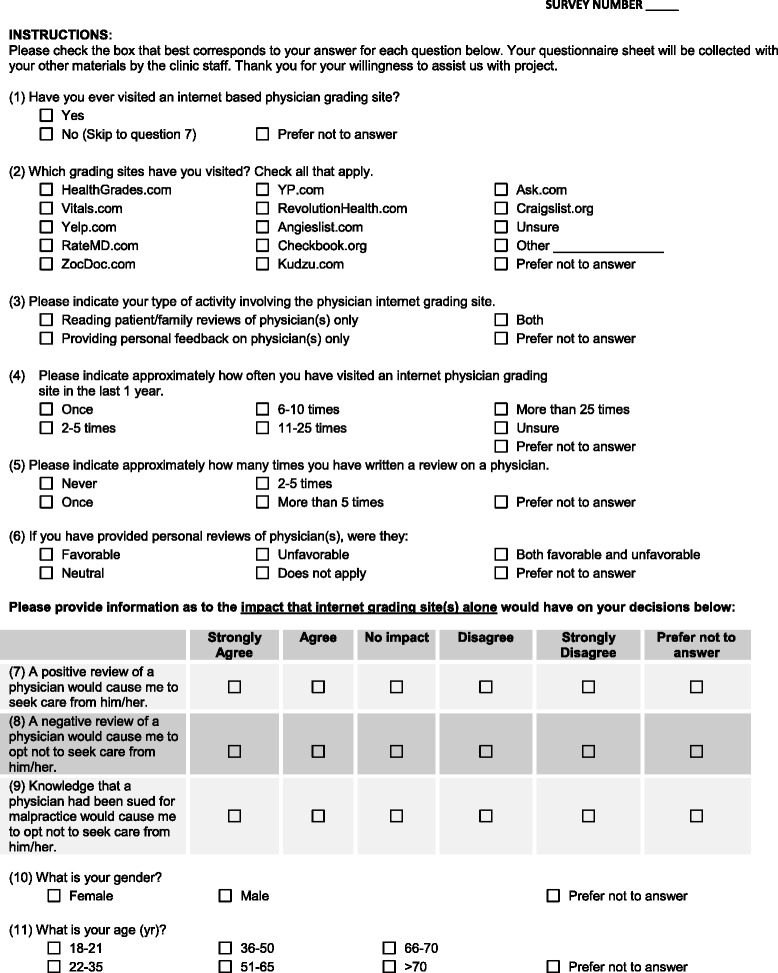
Survey form

Following Mayo Clinic Institutional Review Board approval, 1000 consecutive patients visiting the Pre-Operative Evaluation (POE) Clinic at Mayo Clinic in Rochester Minnesota between June 2013 and October 2013 were surveyed using a written questionnaire (Fig. 1).

Among those patients who verbally consented to participate, survey forms were distributed to as they entered the clinic exam room and returned to clinic personnel at the end of their appointment. Data collected included respondent demographics, whether or not a respondent had visited an IPRS in the past and the frequency with which they had visited, the specific site(s) visited, and the frequency and nature of any comments made. In addition, respondents were asked their level of agreement (on a five point scale) with a series of statements evaluating the influence of IPRS positive or negative reviews or reports of malpractice actions on their decision to seek care from a particular physician. All information provided by study participants was free of patient identifiers and held in strict accordance with anonymity.

### Statistical analysis

Responses which indicated “prefer not to answer” were treated as missing data. Data were analyzed using descriptive statistics and Chi-square and Fisher exact test analyses for comparisons of categorical data with larger and smaller sample sizes, respectively. Responses to questions 7–9 (Fig. 1) were also grouped into three levels, and treated as categorical data for Chi-square and Fisher exact test analyses: “Strongly agree”, “Neutral” (somewhat agree/neutral/somewhat disagree) and “Strongly disagree”. The groupings were defined based on the fact that individuals are likely to seek others only if they hold strong opinions on a subject, as illustrated in the Net Promoter Score [10, 11]. A P-value of less than 0.05 was considered significant.

## Results

A total of 854 respondents completed the survey to some degree for an overall response rate of 85 %. Most respondents (404 of the 837 who provided information on age, 48 %) were between the ages of 51 and 65 years with approximately equal sex distribution (Table 1).

**Table 1:** Demographics of respondents

Demographics		
Sex	Number (838 total)	Percentage of total
Female	429	51.2 %
Male	409	48.8 %
Age (years)	Number (837 total)	Percentage of total
18-21	1	<1 %
22-35	13	1.6 %
36-50	108	12.9 %
51-65	404	48.3 %
66-70	133	15.9 %
>70	178	21.3 %

Respondents who did not provide answers to specific questions – e.g. sex – were excluded from the presentation of data relating to that question

A large majority (714 of 854 respondents, 84 %) of those surveyed stated that they had not visited an IPRS in the past. Of the 140 respondents who had visited a site, a quarter had visited only once in the previous year and half had visited between 2 and 5 times (Table 2).

**Table 2:** Frequency of IPRS visitation among the 140 respondents who stated that they had previously visited a site

Number of IPRS visits over the last year	Number	Percentage of total
Once	35	25.0 %
2-5	76	54.3 %
6-10	15	10.7 %
11-25	5	3.6 %
>25	3	2.1 %
Unsure	6	4.3 %

One hundred thirty eight of the 140 site visitors provided information on their activities. Visits to 10 different sites were reported. Reports of visits were most frequent for Health Grades.com (visited by 45 % of those who named a site), followed by RateMD.com (19 %) and Ask.com (12 %). Thirty percent of respondents were unsure of the IPRS they had used in the past. Most (83 %) had read reviews posted by others rather than writing their own reviews (Table 3).

**Table 3:** Reported IPRS activities among respondents who had visited a site

Type of activity	Number^a^ (138 total)	Percentage of total
Reading prior reviews only	114	82.6 %
Providing written feedback only	6	4.3 %
Both reading reviews and providing written feedback	18	13.0 %

^a^Two of the 140 respondents visiting IPRS failed to provide information on the nature of their activities

Only 17 % of the 140 respondents who had visited a site provided written feedback. This is less than 3 % of the entire surveyed sample. Two respondents had written comments but were unsure of the number, 3 had written once, 12 between 2 and 5 times, and only 1 respondent had commented more than 5 times. Of the commenters, just over a third (36 %) stated that they provided either unfavorable (9 %) or a combination of favorable and unfavorable (27 %) reviews of physician interactions (Table 4).

**Table 4:** Nature of responses among those who had provided written feedback

Type of response provided	Number (22 total)^a^	Percentage of total
Favorable	11	50 %
Neutral	1	4.5 %
Unfavorable	2	9.1 %
Both favorable and unfavorable	6	27.3 %
Prefer not to answer	2	9.1 %

^a^Two of the 24 respondents who reported providing written feedback failed to provide the nature of their responses

Among all respondents, only 28 % strongly agreed that a positive physician review alone on an IPRS would cause them to seek care from that practitioner (Table 5).

**Table 5:** Influence of a positive or negative review and knowledge of malpractice history on decision to seek care

A positive physician review alone would cause me to seek care from that individual	Number (803 total)	Percentage of total
Strongly agree	226	28.1 %
Agree	427	53.2 %
No impact	127	15.8 %
Disagree	14	1.7 %
Strongly disagree	9	1.1 %
A negative physician review alone would cause me not to seek care from that individual	Number (796 total)	Percentage of total
Strongly agree	215	27.0 %
Agree	396	49.7 %
No impact	134	16.8 %
Disagree	41	5.2 %
Strongly disagree	10	1.3 %
Knowledge that a physician had been sued for medical malpractice would cause me not to seek care	Number (790 total)	Percentage of total
Strongly agree	234	29.6 %
Agree	293	37.1 %
No impact	209	26.5 %
Disagree	48	6.1 %
Strongly disagree	6	0.8 %

Similarly, 27 % of those surveyed strongly agreed that a negative IPRS review would cause them to choose against seeking care from that physician. Further, fewer than a third (29.9 %) of respondents indicated that knowledge of a malpractice suit based on IPRS alone would negatively impact their decision to seek care from a physician. Whether a respondent had visited an IPRS in the past had no impact on the decision to seek care. (P values from Chi-square test 0.88, 0.23, 0.30 for positive review, negative review, malpractice information, respectively.)

Twenty three percent of respondents aged ≤ 50 years had visited an IPRS compared with 15 % of those aged over 50 (*P* = 0.03 by Chi-square test). However, there were no significant age-related differences in the responses to any of the other survey questions.

Women were more likely than men to strongly agree with the statement that anegative review or knowledge of a malpractice suit would influence their decision to seek care from a physician, but there were no differences in the level of agreement with the statement regarding a positive review. (Positive review 30 % of women strongly agree versus 26 % of men, *P* = 0.13; negative review 32 % versus 21 %, *P* < 0.01; malpractice knowledge women 36 % versus 22 %, *P* < 0.01).

## Discussion

Our single-center, paper-based survey of pre-surgical patients (with a high response rate of 85 %) found that, despite the widespread use of the Internet in our society, relatively few (16 %) have visited an IPRS. Our findings are consistent with a report by the Pew Research Center that suggested that only 16 % of Internet users, or 12 % of U.S. adults overall, have reviewed online physician or other healthcare provider rating sites [5]. These numbers are also consistent with studies exploring use in other countries. In 2012, Galizzi et al. reported that only 15 % of those they surveyed in the United Kingdom were aware of IPRS [12]. Studies by Emmert at al. and Terlutter and colleagues found a larger percentage of Germans, 32 % and 29 % respectively, were aware of IPRS [13, 14]. Hanauer and colleagues recently found that although 65 % of U.S. respondents in their survey were aware that IPRS existed, this percentage was lower than for other commercial product and service sites [9]. The greater engagement with IPRS in the Hanauer study (36 % of respondents had visited an IPRS at least once, compared with 16 % in our study) might be explained by differences in age profile and Internet familiarity of the study populations.

Although many patients may not be aware of online rating sites, others may use these venues as a source of information to aid decision-making when choosing a physician. It has been suggested that sufficient validation of the information provided on such sites has not yet occurred and that further analysis of the quality and reliability of the information provided is required [7, 9, 15].

Only 24 (17 %) of the 138 respondents in our cohort who had visited IPRS and provided information relating to their activities had written reviews of physicians. This reflects approximately 3 % of all respondents. This small number is consistent with prior reports finding that only 4-5 % of Internet users in the U.S. have posted an online review of their physician in the past [5, 9]. These percentages are much lower than shown by Emmert for German IPRS [16]. Despite the apparently small number of patients who write reviews, some studies have found that a large percentage of physicians have been reviewed. For example, of 500 U.S. urologists randomly selected from a database, approximately 80 % had one or more written reviews posted [7]. Emmert et al. also found that 37 % of all German physicians had been rated in their study [16].

Half of those respondents in our survey that provided written reviews of physicians provided positive comments alone. Another 27 % reported providing both favorable and unfavorable ratings with only 9 % stating that they had provided only unfavorable scores or comments. Concern over the accuracy and reliability of IPRS has been raised by health care professionals, their professional societies and even some state governments [2, 6]. Studies prompted by these concerns have demonstrated that IPRS are, in fact, predominately populated by positive comments rather than those posted by disgruntled patients [3, 6, 8, 9]. Black et al. analyzed over 16,000 ratings of more than 6000 providers and found predominantly high ratings and positive comments [3]. Lagu and colleagues reviewed 33 physician rating sites containing 190 reviews of 81 physicians and found 88 % to contain positive reviews, 6 % negative and another 6 % neutral [8]. More recently, Gao et al., reviewing rating information for 112,000 physicians, found the average rating to be 3.93 out of 5 [6]. When Hanauer et al., surveyed a representative national population base, 54 % of respondents stated that they had provided positive reviews, 29 % neutral reviews with only 19 % reporting negative physician comment [9]. Although these studies illustrate that more positive ratings populate IRPSs than negative ratings, they provide little insight as to the reliability of these data to provide potential patients with beneficial information. The question that remains is whether the ratings and comments offered are due to a core set of prior patients who would populate these IPRS regardless of whether they had a positive or negative experience with a physician or whether they would only sign on to these sites if they had either a positive or a negative comment to make. The former position would indicate better site accuracy and reliability whereas the latter would be biased by motivation on the part of the user of these sites.

Our study found that younger respondents (<50 years of age) were more likely to visit an Internet grading site. This is similar to those reports by Tertlutter et al. who, in an on-line survey of just over 1000 randomly selected German patients, found that younger survey respondents reported greater use of IPRS when compared to older respondents [14]. Emmert and colleagues found that a majority of rating patients in their study fell between the ages of 30 and 50 years of age [13]. Interestingly, older patients were more apt to provide positive patient reviews when compared to their younger counterparts. It has been suggested when the “Facebook and Myspace generations” reach the age when health care needs become of greater importance to them, the popularity and influence that Internet grading sites play may increase [2]. Perhaps consistent with this theory, Terlutter and colleagues found that users of IPRS had a higher digital literacy rate (described as a self -reported level of Internet skills) than non-users [14]. It will be important to explore to what degree such individuals emphasize Internet rating sites when choosing a physician.

A minority of respondents in our study (28 % strongly agreed) reported that a positive physician review would influence their decision to seek care from that practitioner. Similarly, 27 % and 30 %, respectively, strongly agreed that a negative review or knowledge of a malpractice suit against the physician would result in them being reluctant to seek care from that individual. Consistent with the current study, Hanauer reported that only 19 % of respondents to their survey of a representative sample of the United States population mentioned that information available on an IPRS would be very important to their decision to seek or not seek care from an individual physician [9]. The remainder held it to be either somewhat important (40 %) or not important (41 %) in their decision. Our findings are inconsistent with those of Emmert et al. who reported that among the 25 % of those surveyed in Germany who had used an IPRS to search for a physician, 65 % would seek out a physician due to their positive ratings and 52 % would steer away from a physician based on a negative rating profile [13]. These differences in the influence of both positive and negative ratings on physician choice may point to a difference in the perceived reliability of IRPS between US and German patients. This argument is supported by the fact that ourstudy findings are consistent with those described in the US by Hanauer and colleagues.

When comparing the influence that positive ratings, negative ratings and malpractice history might have on a patient’s decision to seek care from that individual, we found no differences in responses among those who had or had not frequented an IPRS in the past. Although only a third of those who report visiting IPRS indicate that they are strongly influenced by the information available and those who do not visit report the same degree of potential influence, any rise in the number of patients that view these sites may have a significant impact on physician choice. Physicians should therefore be aware that if this yet untapped group ends up later frequenting IPRS as their numbers and the available information expand, what they see could have an impact on the care they seek. It is interesting that women seemed to be influenced more than men by negative information on IPRS. Based on our data, postulation of the reasons for a gender-based difference – if one actually exists – would be pure speculation.

A growing concern among practitioners is the reliability of certain rating systems to reflect the level of quality of care provided by those physicians [17, 18]. Published enquiries have largely been limited to seeking the level of correlation between rating scores and hospital quality of care metrics [19, 20]. Greaves at el. found a positive correlation between Internet-based patient ratings and certain objective measures of hospital quality to include infection rates and patient mortality [20]. More recently, high patient ratings of U.S. hospitals provided via Yelp were found to correlate with lower mortality following myocardial infarction (MI) and pneumonia along with fewer readmissions following initial treatment of MI, pneumonia and heart failure [19]. Few studies have explored the interaction between IPRS and physician quality measures however. Gao and colleagues found a weak association between IPRS and certain clinical quality metrics among family practice physicians in England [6]. Fenton et al. showed that patient satisfaction with their health care experience in general (including measures of satisfaction with their physician) among a set of U.S patients was associated with higher overall health care and prescription drug costs as well as increased patient mortality [1]. Given the limited, and often conflicting findings to date, more studies are needed to better determine any correlations that may exist between patient rating scores and physician quality metrics. This information will be increasingly relevant as government and private payers look to base physician reimbursement schedules on patient satisfaction ratings as well as documented quality metrics [17].

There are a number of limitations to our study. Its single center nature may have led to an inability to differentiate institutional reputation from individual clinician reputation. Additionally, we did not collect data on either the level of patient education achievement or their geographic region of origin and are thus unable to evaluate their influence, if any, on the use of IPRS. Previous studies using the same POE Clinic venue have documented a lack of ethnic diversity in our survey population and so it may not adequately reflect minority groups or populations with different beliefs [21]. Galizzi et al. reported in their study that “White British” individuals were less inclined to use IPRS, suggesting that this might be due to prior reports of variations in trust of online sites and concerns for confidentiality among differing socioeconomic groups [12]. In addition, individual survey questions may have been interpreted differently. For example, the respondent may have interpreted “a negative review” of a physician as an isolated negative review or negative reviews in aggregate. Unlike the study performed by Hanauer at all, we limited our patient inquiry to *physician* rating sites alone. This may have biased our patient responses in a way that may not have occurred had we incorporated these same questions into a larger set of product and services Internet rating sites. Further, we asked questions relating only to the influence of information provided in IPRS and not on other factors that may influence a patient’s decision to seek (or not seek) care from a particular decision. This in turn may have biased the influence that IPRS site information may have had for those we queried. Finally, the answers to the hypothetical questions posed may or may not be reflective of actual past or future patient actions.

## Conclusions

In summary, our findings suggest that relatively few patients visit IPRS and the influence of site-derived information on their decision to seek (or not seek) care from a particular physician appears to be limited. Further, among the small number of patients who do provide feedback on IPRS, our findings show that a majority of ratings were positive. It remains unknown, however, to what degree there is a true association between physician rating scores and actual quality of medical services provided.
